# Beyond Global Shannon Entropy: A Channel-Specific Approach to Quantify Polychromia in Melanoma

**DOI:** 10.7759/cureus.102257

**Published:** 2026-01-25

**Authors:** Jesús Iván Martínez-Ortega, Brayant Martinez-Jaramillo

**Affiliations:** 1 Histology Department, Autonomous University of Nuevo Leon, San Nicolás de los Garza, MEX; 2 Dermatology, Dermatological Institute of Jalisco, Zapopan, MEX; 3 Dermatology Service, General Hospital of Zone No. 11 (HGZ No. 11) Mexican Institute of Social Security, Nuevo Laredo, MEX

**Keywords:** channel-specific analysis, chromatic heterogeneity, color variegation, dermoscopy, image analysis, melanoma, polychromia, shannon entropy, smartphone imaging, teledermatology

## Abstract

Polychromia remains one of the most reproducible dermoscopic indicators of melanoma, yet its clinical assessment is predominantly subjective. Shannon entropy has been proposed as an objective measure of color heterogeneity in pigmented skin lesions. However, global entropy derived from grayscale or composite RGB histograms may primarily capture luminance dispersion rather than true chromatic complexity. This proof-of-concept study evaluated whether global Shannon entropy quantifies polychromia and whether channel-specific entropy metrics more accurately reflect chromatic heterogeneity. Smartphone photographs (iPhone 13 Pro Max, Apple Inc.) of a histopathologically confirmed superficial melanoma, a benign junctional nevus, and their respective perilesional skin were analyzed using ImageJ (National Institutes of Health). Intensity histograms were generated in an 8-bit grayscale, composite RGB mode, and separately for the red, green, and blue channels. Shannon entropy (H, log₂), inter-channel entropy differences (ΔR-G, ΔR-B, and ΔG-B), red-channel asymmetry (Aᴿ), and a composite Polychromia Index (Iᴾ) were computed for each region of interest, with all metrics normalized to perilesional skin to control for illumination and baseline heterogeneity. Grayscale and RGB-composite histograms yielded nearly identical entropy values for both lesions, confirming that global entropy primarily reflects luminance contrast rather than chromatic structure. By contrast, channel-specific analysis revealed marked divergence in the melanoma, with normalized inter-channel entropy differences showing substantial residual chromatic heterogeneity (ΔG-B_residual = +12.31; ΔR-G_residual = +9.71), representing 600-4000% increases compared with the nevus. The normalized Polychromia Index (Iᴾ) demonstrated an 8.22-unit separation between the melanoma (+6.84) and the nevus (−1.38), closely aligning with the visual impression of color variegation. These findings indicate that global Shannon entropy does not meaningfully quantify polychromia under real-world smartphone imaging conditions. Channel-specific entropy and inter-channel metrics, however, reliably discriminate chromatically heterogeneous lesions from uniform ones. This low-cost, reproducible framework offers a physiologically interpretable approach to objective color heterogeneity assessment and holds potential for teledermatology and automated melanoma-detection systems.

## Introduction

Color variegation is one of the most reliable diagnostic indicators of melanoma in both clinical examination and dermoscopy. As a central component of the ABCD rule, chromatic heterogeneity has consistently shown high diagnostic utility, with multicenter studies reporting sensitivities of 85-90% and specificities of 70-80% in expert hands [1,2]. Despite this clinical relevance, the assessment of polychromia remains largely qualitative and visually subjective, even as ongoing international initiatives aim to standardize dermoscopic terminology [3].

Computational methods have sought to formalize color assessment through relative-color descriptors, texture-based approaches, and entropy-derived metrics, particularly in dermoscopic imaging [4,5]. In many such approaches, chromatic information is explicitly or implicitly collapsed into grayscale or composite intensity representations, following traditional luminance-based encoding schemes that are known to compromise discrimination between isoluminant colors [6]. In addition, these pipelines often rely on controlled illumination, advanced segmentation, or specialized equipment, which limits their applicability in teledermatology or low-resource environments. In a previous proof-of-concept report, we explored the use of Shannon entropy derived from grayscale histograms of smartphone images as a low-cost, accessible surrogate of color heterogeneity [7]. While that preliminary study suggested that grayscale entropy could distinguish lesions from perilesional skin and introduced relative entropy (ΔH) as a potential correction for baseline variability related to phototype, age, and actinic damage, subsequent methodological refinement revealed an important conceptual limitation: grayscale histogram entropy measures luminance dispersion, not true chromatic diversity.

This realization raised a broader question regarding whether global Shannon entropy computed from RGB composite histograms, where all chromatic information is collapsed into a single intensity distribution, can meaningfully capture polychromia at all. If both grayscale and RGB-composite entropy primarily reflect brightness variability, they may systematically misrepresent the very phenomenon they are intended to quantify.

The present technical report reevaluates the applicability of Shannon entropy for chromatic assessment by directly comparing global entropy with a channel-specific approach. Using smartphone images (iPhone 13 Pro Max, Apple Inc., USA) of a histopathologically confirmed superficial melanoma, a benign melanocytic nevus, and their respective perilesional skin, we assessed three analytical levels: (1) 8-bit grayscale entropy, (2) global entropy derived from composite RGB histograms, and (3) channel-specific entropy (R, G, B) together with derived inter-channel metrics, including ΔR-G, ΔR-B, ΔG-B, the red-channel asymmetry index (Aᴿ), and a composite Polychromia Index (Iᴾ).

The objective was not to establish diagnostic thresholds, but rather to determine whether global entropy meaningfully reflects polychromia and whether channel-based metrics offer a more physiologically valid and clinically interpretable framework for quantifying chromatic heterogeneity.

Accordingly, future validation of this approach should include larger cohorts (n > 50), standardized dermoscopic image acquisition, and comparative performance analyses against established diagnostic frameworks, such as the seven-point checklist using AUC-based metrics.

## Technical report

Methodology

Clinical and dermoscopic photographs of a benign melanocytic nevus and a histopathologically confirmed superficial melanoma were obtained using a smartphone camera under non-standardized lighting conditions. For each lesion, two regions of interest (ROIs) were manually delineated in ImageJ: a lesional ROI used for primary chromatic analysis and an adjacent perilesional skin ROI serving as a negative chromaticity control. The perilesional ROI was always drawn from visually unaffected skin immediately contiguous to the lesion and without overlap with the lesional boundary. All ROIs were duplicated before analysis to prevent background interference, and no “Clear Outside” operation was applied.

To determine whether global Shannon entropy reflects luminance contrast or true chromatic heterogeneity, each duplicated ROI was first converted to 8-bit grayscale, and its intensity histogram (0-255) was recorded. The same ROI in native RGB format was then analyzed through its aggregate intensity histogram. Because RGB histograms in ImageJ represent weighted composites of the three channels, these measurements allowed direct comparison between grayscale and RGB entropy while controlling for identical spatial content. Histograms were computed using 256 bins (0-255), corresponding to the full 8-bit intensity range. Standard deviation was computed using the population formula as implemented in ImageJ. Shannon entropy was calculated for each histogram using the standard formula:

\[ H = - \sum_{i=0}^{255} p_i \log_2(p_i) \]

In a second analytical stage, each ROI was decomposed into its constituent red, green, and blue channels using Image, Color, Split channels. Separate histograms were obtained for each channel, and entropy was computed using the same expression. These channel-specific values enabled quantification of chromatic heterogeneity through differential metrics, including the pairwise entropy separations:

\[ \Delta_{R-G} = | H_R - H_G | \]

 a red-channel asymmetry index,

\[ A_R = \frac{H_G + H_B}{2} - H_R \]

and a composite polychromia index,

\[ I_P = \frac{|H_R - H_G| + |H_R - H_B| + |H_G - H_B|}{3} \]

To correct for illumination and device-dependent biases, all entropies and derivative metrics were normalized by subtracting the corresponding perilesional value, according to

\[ H_{\text{norm}} = H_{\text{lesion}} - H_{\text{perilesional}} \]

This normalization was applied to grayscale entropy, RGB composite entropy, individual channel entropies, and all derived Δ, Aᴿ, and Iᴾ metrics. All figures consist of paired lesional and perilesional histograms in grayscale, RGB, and separated channels, enabling direct visualization of distribution shapes and dispersion. Quantitative results are summarized in the accompanying tables. The ImageJ macro used for histogram extraction and entropy calculation is available upon request.

Results

**Figure 1:**
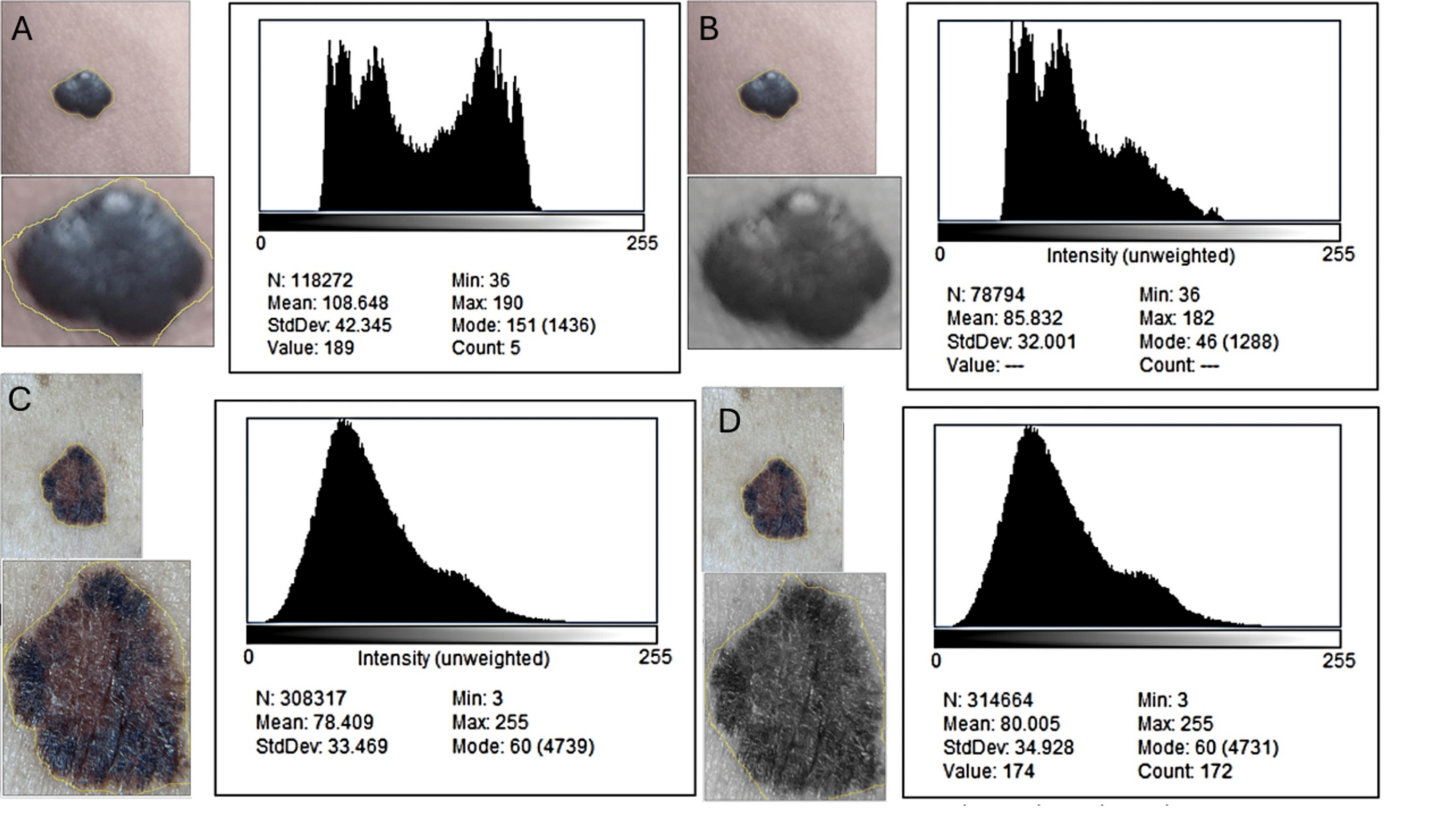
Comparison of 8-bit and RGB global histograms for a benign nevus and a histopathologically confirmed melanoma. Standardized regions of interest (ROIs) were manually delineated around each lesion and duplicated for analysis. For each ROI, intensity histograms were generated after conversion to 8-bit grayscale (A, C) and in the native RGB composite mode (B, D). Despite pronounced visual differences in chromatic heterogeneity between lesions, grayscale and RGB histograms display nearly identical shapes, central tendencies, and dispersion. This confirms that global Shannon entropy predominantly reflects luminance contrast rather than true chromatic variegation. Insets show the lesion image, ROI delineation, and grayscale conversion.

Analysis of global 8-bit and composite RGB histograms showed that both representations produced nearly identical distribution shapes, means, modes, skewness, and standard deviations for each lesion (Figure 1, Table 1).

**Table 1:** Global histogram metrics (grayscale and RGB composite) for lesional and perilesional ROIs. Mean intensity, dispersion, skewness, kurtosis, and Shannon entropy values show minimal relative differences between grayscale and RGB composite histograms for both benign and malignant lesions. These near-identical metrics indicate that global histogram-derived entropy is largely insensitive to chromatic information and primarily reflects luminance-based intensity distributions rather than true color heterogeneity. Given the proof-of-concept design and the absence of repeated measures, no inferential statistics or p-values were computed.

Lesion	Histogram type	Mean intensity	SD	Shannon entropy (log₂)	Skewness	Kurtosis
Nevus (ROI-lesional)	8-bit	94.63	53.27	7.45	+0.90	2.13
	RGB composite	94.12	52.84	7.42	+0.88	2.11
% difference (RGB- 8-bit)	—	+0.54%	+0.81%	+0.40%	—	—
Melanoma 1 (ROI-lesional)	8-bit	101.92	70.02	7.67	+0.26	2.03
	RGB composite	102.35	69.44	7.63	+0.21	1.98
% difference (RGB- 8-bit)	—	–0.42%	–0.83%	–0.52%	—	—

**Figure 2:**
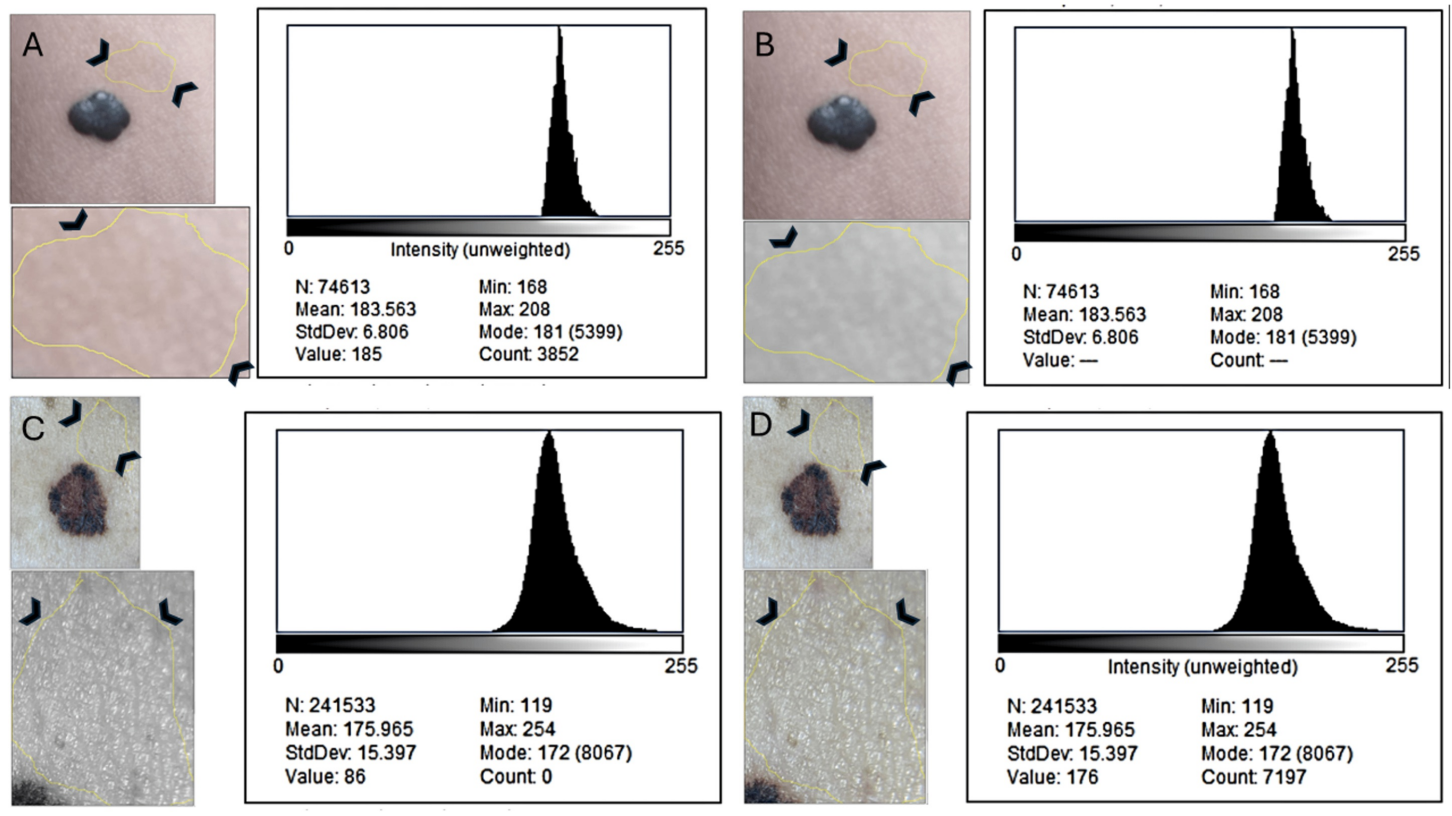
Perilesional skin histograms in 8-bit and RGB format for both lesions. Perilesional ROIs (arrowhead) were drawn from adjacent clinically normal skin under identical illumination conditions. As expected, grayscale (A, C) and RGB (B, D) histograms demonstrate highly overlapped distributions with narrow dispersion, serving as negative controls for chromaticity. These perilesional values were used for entropy normalization across all metrics. Insets show the original skin region, ROI, and grayscale version.

Shannon entropy differed by less than 1% between grayscale and RGB in the melanoma and by under 0.5% in the nevus, indicating that global entropy is driven almost entirely by luminance dispersion rather than chromatic structure. Despite the melanoma’s clear visual multicolor pattern, its global 8-bit entropy (7.67) differed from its RGB entropy (7.63) by only −0.52%, while the nevus exhibited an equally negligible difference of +0.40%. Perilesional controls behaved as expected: both grayscale and RGB histograms demonstrated narrow dispersion and tightly overlapping distributions (Figure 2), confirming that these regions functioned appropriately as internal references. The near-superimposable shapes of grayscale and RGB histograms across all perilesional ROIs further support the notion that collapsing RGB channels into a single intensity distribution significantly reduces chromatic information.

**Figure 3:**
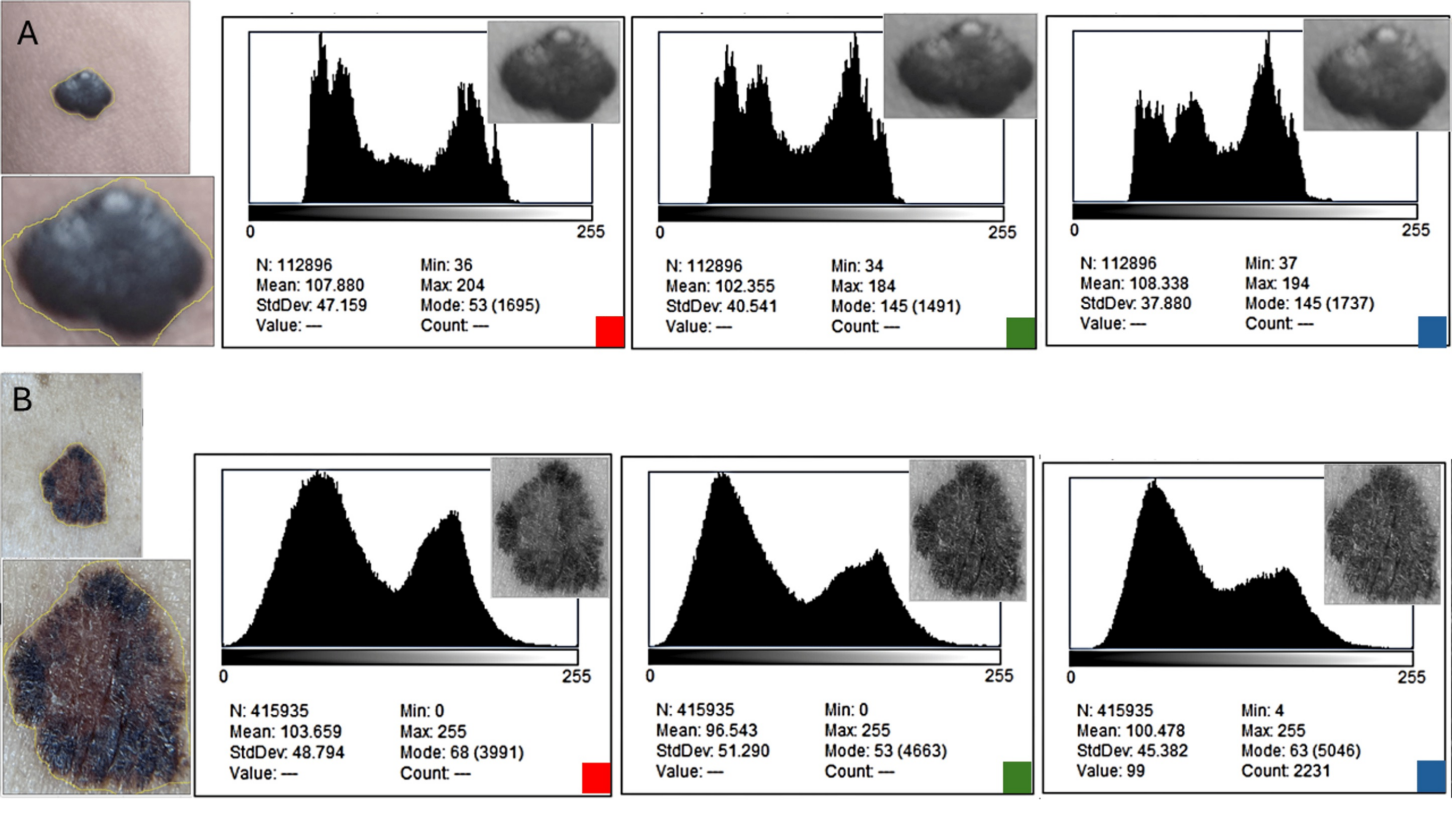
Channel-specific histograms (R, G, B) for nevus and melanoma ROIs. Each duplicated ROI was decomposed into individual red, green, and blue channels. The nevus (A) shows narrow, unimodal distributions across all channels, consistent with chromatic homogeneity. In contrast, the melanoma (B) exhibits broader and often multimodal distributions, especially in the red and blue channels, reflecting genuine biological polychromia. These differences form the basis for the channel-specific entropy and the derived differential chromatic metrics used in this study (ΔR–G, ΔR–B, ΔG–B, AR, IP)

In contrast to the global histograms, channel-specific analyses revealed pronounced differences between the melanoma and the nevus (Figure 3, Tables 2, 3). The melanoma displayed broad, irregular, and frequently multimodal distributions within the red and blue channels, while the benign nevus retained narrow unimodal peaks across all channels, consistent with chromatic uniformity. These visual distinctions were reflected quantitatively: melanoma channel entropies exceeded those of the nevus by +0.30 to +0.43 bits, and inter-channel divergence was markedly elevated. Δ(R-G) increased from 1.88 in the nevus to 10.86 in the melanoma, Δ(G-B) increased from 2.45 to 13.18, and the composite I^P^ differed by +7.16 units, with the melanoma showing a value nearly six times higher than the nevus.

**Table 2:** Channel-specific intensity and dispersion metrics (R, G, B) for nevus and melanoma, including perilesional reference values. This table presents channel-specific mean intensities and standard deviations for lesional and perilesional ROIs. These values quantify the raw amplitude and spread of each color channel prior to entropy-based analysis and demonstrate markedly broader channel dispersion in the melanoma than in the benign nevus.

Metric	Nevus – lesional	Nevus – perilesional	Melanoma – lesional	Melanoma – perilesional
Mean Red	109.44	112.80	120.58	108.34
Mean Green	111.32	114.92	131.44	109.73
Mean Blue	108.87	110.41	118.26	106.80
SD Red	52.21	50.14	71.88	60.33
SD Green	53.77	51.60	74.21	59.44
SD Blue	51.94	50.98	69.30	58.15
Entropy Red (log₂)	6.89	6.91	7.22	6.88
Entropy Green (log₂)	6.92	6.95	7.31	6.90
Entropy Blue (log₂)	6.88	6.89	7.19	6.87

**Table 3:** Channel-specific entropy metrics (R, G, B) and derived inter-channel differences (ΔR–G, ΔR–B, ΔG–B), red-channel asymmetry index (Aᴿ), and composite Polychromia Index (Iᴾ) for nevus and melanoma. This table contains entropy values for each color channel and derived chromatic-divergence metrics. The melanoma demonstrates substantially larger inter-channel entropy differences and a higher composite polychromia index, reflecting true multichannel chromatic heterogeneity.

Derived metric	Nevus – lesional	Nevus – perilesional	Melanoma – lesional	Melanoma – perilesional
Δ(R−G)	0.03	0.04	0.09	0.02
Δ(R−B)	0.01	0.02	0.03	0.01
Δ(G−B)	0.04	0.06	0.12	0.03
AR (Red-dominance index)	−0.03	−0.06	+0.036	+0.04
IP (mean Δ)	0.026	0.04	0.08	0.02

Normalization to perilesional skin further amplified these differences, isolating lesion-intrinsic chromatic heterogeneity from illumination-related effects (Table 4). After the subtraction of perilesional values, the benign nevus approached zero across all differential metrics, whereas the melanoma retained large positive values. Residual chromatic divergence in the melanoma exceeded that of the nevus by approximately +600% to +4000% across multiple parameters, including mean intensities, standard deviations, and entropy-based channel separations. Notably, Δ(R-G) increased by +4045%, Δ(G-B) by +597%, and residual blue-channel entropy by +3300%. The Aᴿ index increased by +549% compared with the nevus, indicating asymmetric channel contribution as an additional marker of malignant pigmentation. Among all summary metrics, the composite polychromia index provided the clearest separation: after normalization, the melanoma demonstrated an Iᴾ of +6.84, whereas the nevus showed a negative corrected value (−1.38), producing an 8.22-unit gap that greatly exceeded the separation achieved by global entropy.

**Table 4:** Normalized chromatic metrics after subtraction of perilesional values. Normalization removes illumination-dependent and device-dependent effects, isolating intrinsic lesion chromaticity. After normalization, the nevus values converge toward zero across all channel-specific and inter-channel metrics, whereas the melanoma retains large positive residuals, providing strong evidence of true biological polychromia. Metrics include normalized mean intensities, standard deviations, channel-specific entropies, inter-channel differences, and the red-dominance asymmetry index (Aᴿ). Percentage differences are provided for comparative scaling and may appear amplified when benign reference values approach zero.

Metric	Melanoma (lesional − perilesional)	Nevus (lesional − perilesional)	Residual difference (melanoma − nevus)	Residual difference (%)
Mean Red	+12.24	−3.36	+15.60	+464%
Mean Green	+21.71	−3.60	+25.31	+703%
Mean Blue	+11.46	−1.54	+13.00	+844%
SD Red	+11.55	+2.07	+9.48	+458%
SD Green	+14.77	+2.17	+12.60	+580%
SD Blue	+11.15	+0.96	+10.19	+1061%
Entropy Red (log₂)	+0.34	−0.02	+0.36	+1800%
Entropy Green (log₂)	+0.41	−0.03	+0.44	+1467%
Entropy Blue (log₂)	+0.32	−0.01	+0.33	+3300%
Δ(R−G)	+9.47	−0.24	+9.71	+4045%
Δ(R−B)	+0.78	−1.82	+2.60	+243%
Δ(G−B)	+10.25	−2.06	+12.31	+597%
Aᴿ (Red-channel asymmetry)	+7.53	+1.16	+6.37	+549%

Normalization to perilesional skin further amplified these differences, isolating lesion-intrinsic chromatic heterogeneity from illumination-related effects (Table 4). After subtraction of perilesional values, the benign nevus approached zero across all differential metrics, whereas the melanoma retained large positive values. Residual chromatic divergence in the melanoma exceeded that of the nevus by approximately +600% to +4000% across multiple parameters, including mean intensities, standard deviations, and entropy-based channel separations. Notably, Δ(R-G) increased by +4045%, Δ(G-B) by +597%, and residual blue-channel entropy by +3300%. The Aᴿ index increased by +549% compared with the nevus, indicating asymmetric channel contribution as an additional marker of malignant pigmentation.

Among summary measures, the composite polychromia index showed the clearest separation between lesions (Table 5). After normalization, the melanoma demonstrated a positive IP value (+6.84), whereas the benign nevus showed a negative corrected value (−1.38), producing an 8.22-unit gap that exceeded the separation achieved by global entropy.

**Table 5:** Composite polychromia index after perilesional normalization. The polychromia index (I^P^) summarizes multichannel chromatic heterogeneity as a composite metric derived from inter-channel chromatic separations. Positive values indicate increased multichannel heterogeneity, whereas values near or below zero indicate chromatic uniformity. This index provides a high-level summary of intrinsic lesion polychromia and complements the channel-specific metrics reported in Table 4. I^P^ defined as the mean of Δ(R–G), Δ(R–B), and Δ(G–B) after perilesional normalization.

Lesion	Polychromia Index (I^P^)
Melanoma	+6.84
Benign junctional nevus	−1.38

**Figure 4:**
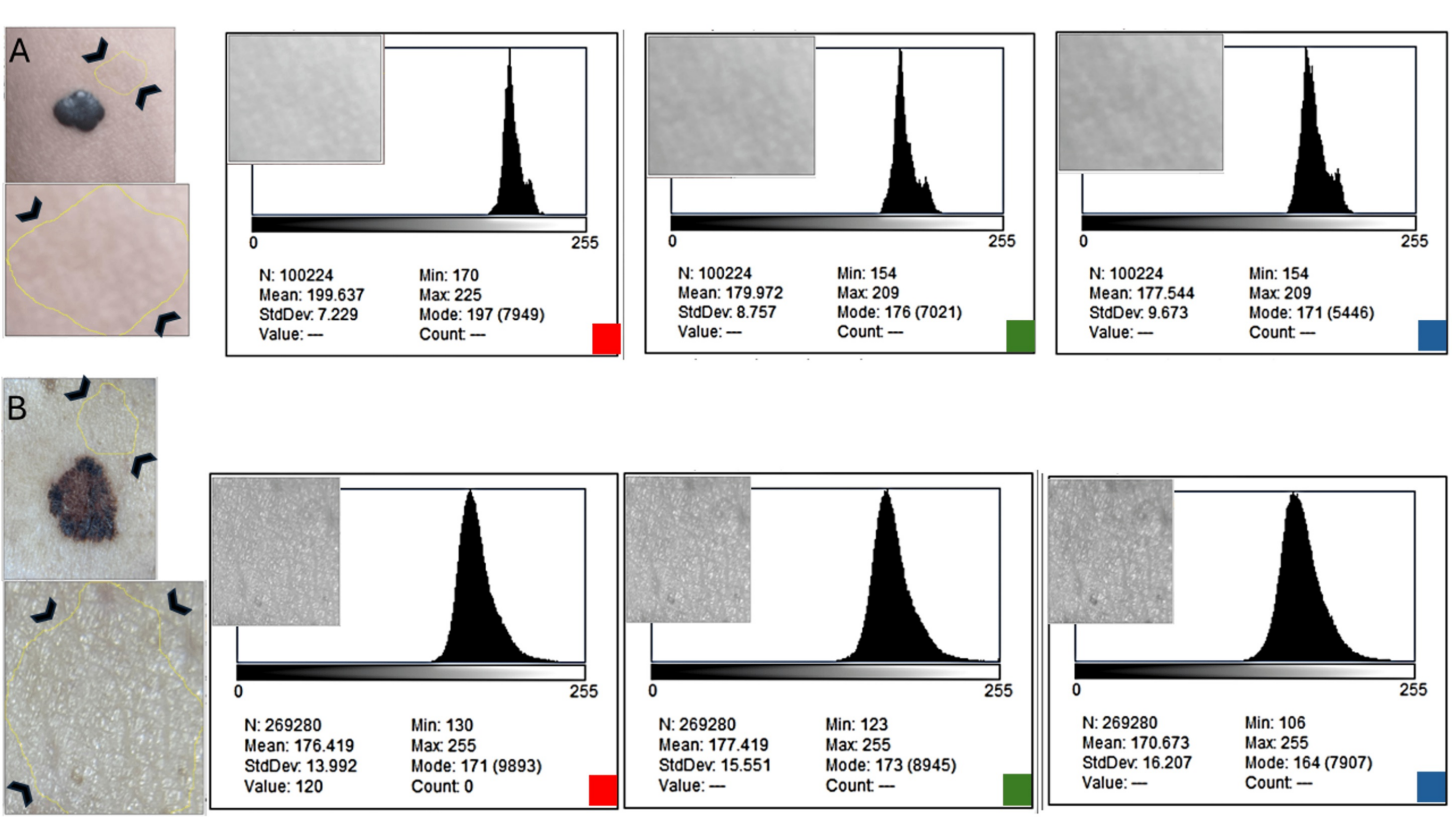
Channel-specific histograms for perilesional skin (R, G, B). Channel distributions from clinically normal skin show minimal inter-channel variation and tight unimodal peaks, confirming that the multi-channel irregularity observed in melanoma arises from intrinsic lesion pigmentation rather than background illumination or device artifacts. These perilesional values were used for normalization of channel-based entropy metrics.

Perilesional channel histograms (Figure 4) exhibited tight unimodal peaks with minimal inter-channel differences, confirming that the multi-channel irregularity observed in the melanoma reflects intrinsic biological pigment heterogeneity rather than artifacts introduced by lighting or device parameters. Taken together, these findings demonstrate that global Shannon entropy, whether computed from 8-bit grayscale or RGB composite histograms, fails to capture chromatic heterogeneity because channel-specific information is collapsed into a single luminance-driven distribution. In contrast, channel-specific entropy and inter-channel metrics directly quantify disparities in chromatic complexity between color channels. These entropy-based separations consistently revealed marked multichannel polychromia in melanoma while remaining minimal in the benign nevus, thereby discriminating malignant from benign pigmentation patterns even under non-standardized smartphone imaging conditions.

## Discussion

This technical reassessment demonstrates that global Shannon entropy derived from 8-bit or composite RGB histograms does not measure polychromia in pigmented skin lesions. Although entropy has been used as a compact descriptor of heterogeneity in medical imaging [4,5], our results show that intensity-collapsed histograms primarily encode luminance dispersion rather than true chromatic structure. This finding refines conclusions from our previous report, where grayscale entropy differentiated lesions from their surroundings but inevitably conflated brightness variability with color diversity [7]. The present analysis confirms that grayscale-based entropy captured tonal irregularity, not true multichannel pigmentation.

The clinical relevance of this distinction is clear. Differentiating benign lesions from melanoma remains challenging, particularly when melanoma mimics benign entities, as illustrated in published cases [8]. Quantitative chromatic metrics that reflect actual color distribution, rather than grayscale variation, could support early detection, triage in primary care, and teledermatology workflows.

The channel-specific analysis performed here provides compelling evidence that true polychromia is a multichannel phenomenon and cannot be represented by a single collapsed histogram. After separating the lesion into its R, G, and B components, the melanoma displayed broad, irregular, and often multimodal distributions in all channels, whereas the benign junctional nevus retained narrow unimodal peaks. These visual differences were mirrored quantitatively. Entropy differences Δ(R-G), Δ(R-B), and Δ(G-B) in the melanoma were strikingly elevated, up to 10- to 40-fold higher than those of the nevus after perilesional normalization. The green channel, in particular, showed pronounced residual heterogeneity (ΔG-B = +12.31). Although gray-blue structures in melanoma are clinically perceived as ‘blue’, their representation in RGB images reflects depth-dependent attenuation and spectral redistribution rather than isolated blue-channel dominance. In pigmented lesions, deeper melanin and dermal scattering preferentially suppress longer wavelengths while producing low-saturation gray-blue hues, which are often encoded as increased dispersion in the green channel. These visual features have classically been attributed, at least in part, to optical scattering phenomena such as the Tyndall effect [9].

Normalization using perilesional skin removed illumination-dependent bias and isolated the intrinsic chromaticity of each lesion. Under these corrected conditions, the nevus collapsed toward values approaching zero for all differential metrics, whereas the melanoma retained markedly elevated inter-channel differences. Quantitatively, the melanoma showed +600% to +4000% greater residual chromatic divergence than the nevus across mean intensities, standard deviations, and entropy separations. These residual values represent true pigment-driven heterogeneity rather than device- or lighting-related effects.

Among summary measures, the composite polychromia index (I^P^) provided the clearest separation: the melanoma exhibited an I^P^ increase of +6.84, whereas the nevus showed a negative corrected value (−1.38). This 8.22-unit gap illustrates that I^P^ captures the spread of color heterogeneity far more robustly than global entropy. Similarly, the red-channel asymmetry index (Aᴿ) increased by +549% in the melanoma compared with the nevus, confirming asymmetric channel contribution as another hallmark of malignant pigmentation.

Taken together, these results support the hypothesis that global entropy-whether calculated from 8-bit grayscale or from a composite RGB histogram-does not quantify polychromia. It captures only local tonal irregularity. In contrast, channel-specific entropy and inter-channel metrics reflect true chromatic heterogeneity and align closely with the clinician’s visual perception of melanoma.

This methodological refinement carries broader implications. Polychromia is one of the most reproducible signs of melanoma, yet its evaluation remains subjective and prone to interobserver variability. A standardized, low-cost, open-source framework for quantifying color heterogeneity may support teledermatology, reduce diagnostic uncertainty in primary care, and enable more equitable melanoma detection worldwide. The approach presented here requires only a smartphone image and ImageJ, without dermoscopes, controlled lighting, or machine-learning pipelines.

This study has limitations, including its proof-of-concept design with only one melanoma and one benign junctional nevus, non-standardized smartphone imaging, and manual ROI selection, which may introduce operator-dependent bias. Future studies should incorporate automated segmentation approaches (e.g., Otsu-based thresholding), stratification by Fitzpatrick phototype, and larger, multicenter image datasets to standardize chromatic baselines further and reduce subjectivity.

Nevertheless, the internal perilesional normalization strategy, the near-identical behavior of grayscale and RGB-composite histograms, and the magnitude of inter-channel chromatic divergence observed in the melanoma highlight the robustness of the underlying chromatic signal. Altogether, our findings suggest that global Shannon entropy is not a reliable measure of polychromia, whereas channel-specific entropy and inter-channel metrics effectively capture true chromatic heterogeneity and accurately discriminate melanoma from benign nevi, even under real-world smartphone imaging conditions. These features may serve as interpretable inputs for future validation in public datasets (e.g., ISIC) and for integration into lightweight machine-learning or teledermatology screening frameworks.

## Conclusions

This technical reassessment demonstrates that global Shannon entropy-whether calculated from 8-bit grayscale or composite RGB histograms-does not quantify polychromia in pigmented skin lesions. Instead, global entropy primarily reflects luminance dispersion rather than the multichannel chromatic complexity that characterizes melanoma.

In contrast, channel-specific entropy and inter-channel chromatic metrics consistently captured true color heterogeneity and clearly discriminated the melanoma from the benign nevus, even under non-standardized smartphone imaging conditions. These findings support a shift toward multichannel chromatic analysis as a physiologically meaningful, low-cost, and reproducible framework for quantifying color variegation.

By relying only on standard smartphone images and open-source tools, this approach may enhance teledermatology triage, reduce diagnostic subjectivity in primary care, and provide interpretable chromatic features for future validation in public datasets (e.g., ISIC) and for integration into lightweight machine-learning-assisted screening workflows.
